# Supplementary material for: Novel Driver Strength Index highlights important cancer genes in TCGA PanCanAtlas patients
Source: PeerJ. 2022 Aug 11;10:e13860. doi: 10.7717/peerj.13860 (PMC9375969; doi:10.7717/peerj.13860)
Supplement: Supplemental Information 2 [file peerj-10-13860-s002.zip › COHORTS/genes plots/detailed/2021_8_16_14_9_distribution_events_detailed_males_17.pdf]

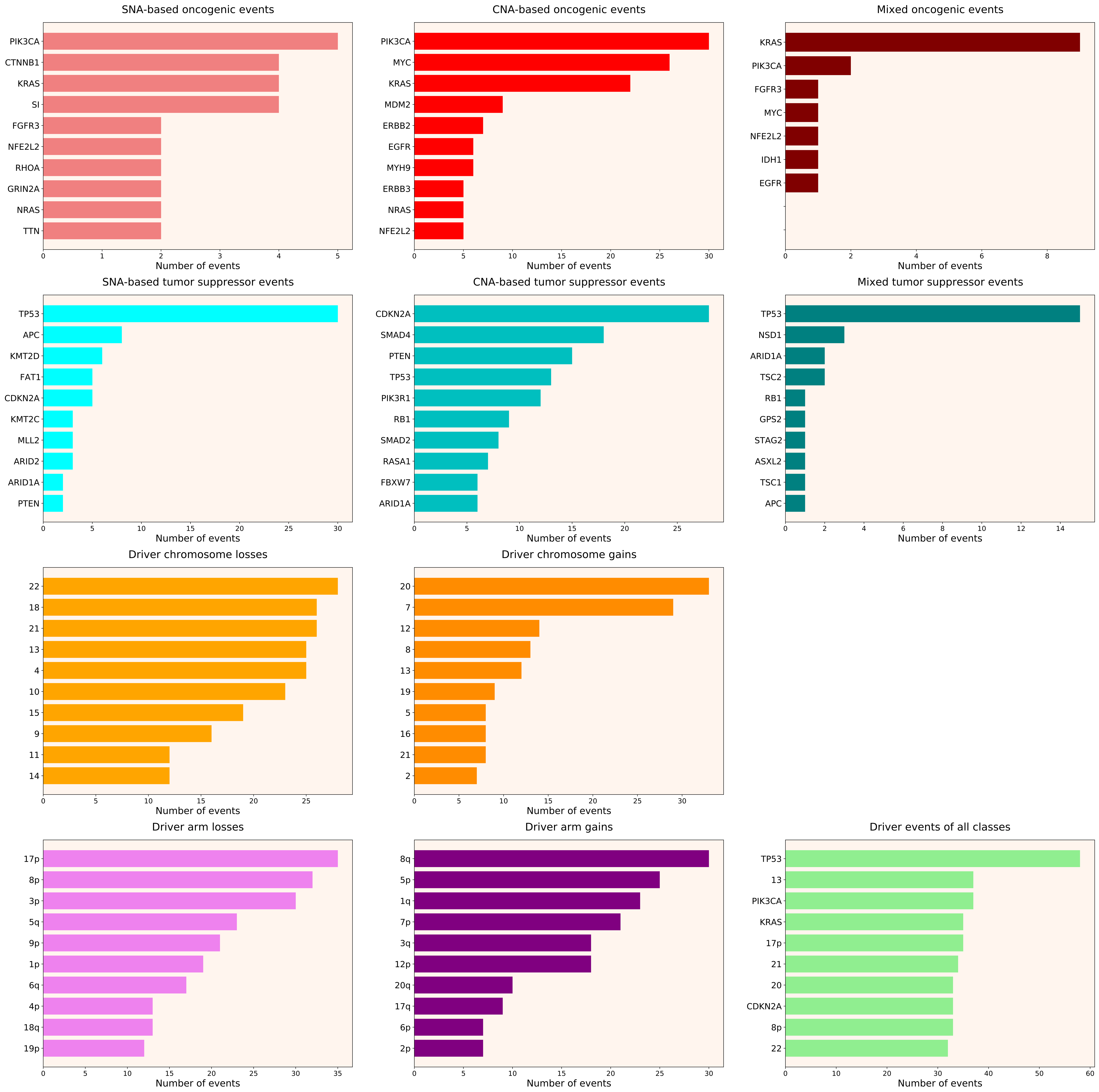

SNA-based oncogenic events
PIK3CA
CTNNB1
KRAS
SI
FGFR3
NFE2L2
RHOA
GRIN2A
NRAS
TTN
Number of events
CNA-based oncogenic events
PIK3CA
MYC
KRAS
MDM2
ERBB2
EGFR
MYH9
ERBB3
NRAS
NFE2L2
Number of events
Mixed oncogenic events
KRAS
PIK3CA
FGFR3
MYC
NFE2L2
IDH1
EGFR
Number of events
SNA-based tumor suppressor events
TP53
APC
KMT2D
FAT1
CDKN2A
KMT2C
MLL2
ARID2
ARID1A
PTEN
Number of events
CNA-based tumor suppressor events
CDKN2A
SMAD4
PTEN
TP53
PIK3R1
RB1
SMAD2
RASA1
FBXW7
ARID1A
Number of events
Mixed tumor suppressor events
TP53
NSD1
ARID1A
TSC2
RB1
GPS2
STAG2
ASXL2
TSC1
APC
Number of events
Driver chromosome losses
22
18
21
13
4
10
15
9
11
14
Number of events
Driver chromosome gains
20
7
12
8
13
19
5
16
21
2
Number of events
Driver arm losses
17p
8p
3p
5q
9p
1p
6q
4p
18q
19p
Number of events
Driver arm gains
8q
5p
1q
7p
3q
12p
20q
17q
6p
2p
Number of events
Driver events of all classes
TP53
13
PIK3CA
KRAS
17p
21
20
CDKN2A
8p
22
Number of events
